# Who Delivers without Water? A Multi Country Analysis of Water and Sanitation in the Childbirth Environment

**DOI:** 10.1371/journal.pone.0160572

**Published:** 2016-08-17

**Authors:** Giorgia Gon, María Clara Restrepo-Méndez, Oona M. R. Campbell, Aluísio J. D. Barros, Susannah Woodd, Lenka Benova, Wendy J. Graham

**Affiliations:** 1 Infectious Disease Epidemiology Department, London School of Hygiene and Tropical Medicine, London, UK; 2 International Center for Equity in Health, Federal University of Pelotas, Pelotas, Brazil; Institute for Health & the Environment, UNITED STATES

## Abstract

**Background and Objectives:**

Hygiene during childbirth is essential to the health of mothers and newborns, irrespective of where birth takes place. This paper investigates the status of water and sanitation in both the home and facility childbirth environments, and for whom and where this is a more significant problem.

**Methods:**

We used three datasets: a global dataset, with information on the home environment from 58 countries, and two datasets for each of four countries in Eastern Africa: a healthcare facility dataset, and a dataset that incorporated information on facilities and the home environment to create a comprehensive description of birth environments in those countries. We constructed indices of improved water, and improved water and sanitation combined (WATSAN), for the home and healthcare facilities. The Joint Monitoring Program was used to construct indices for household; we tailored them to the facility context–household and facility indices include different components. We described what proportion of women delivered in an environment with improved WATSAN. For those women who delivered at home, we calculated what proportion had improved WATSAN by socio-economic status, education and rural-urban status.

**Results:**

Among women delivering at home (58 countries), coverage of improved WATSAN by region varied from 9% to 53%. Fewer than 15% of women who delivered at home in Sub-Saharan Africa, had access to water and sanitation infrastructure (range 0.1% to 37%). This was worse among the poorest, the less educated and those living in rural areas. In Eastern Africa, where we looked at both the home and facility childbirth environment, a third of women delivered in an environment with improved water in Uganda and Rwanda; whereas, 18% of women in Kenya and 7% in Tanzania delivered with improved water and sanitation. Across the four countries, less than half of the facility deliveries had improved water, or improved water and sanitation in the childbirth environment.

**Conclusions:**

Access to water and sanitation during childbirth is poor across low and middle-income countries. Even when women travel to health facilities for childbirth, they are not guaranteed access to basic WATSAN infrastructure. These indicators should be measured routinely in order to inform improvements.

## Background

Hygiene at the time of birth is important to the health of mothers and newborns, irrespective of whether childbirth takes place at home or in a facility. Existing studies link neonatal sepsis and maternal mortality to poor access to water and sanitation (WATSAN)–essential for hygiene practices, in both environments.[1–4] Moreover, historical evidence strongly links maternal mortality and hygiene at birth in facilities.[5–7] Birth-related infections cause the death of many mothers and babies. Infection contributes to at least 9% of maternal deaths, and 680 000 neonatal deaths annually; these are concentrated in low and middle-income countries (LMICs) and are likely to be underestimates.[8,9] Indeed, the rate of newborn infections among babies born in hospitals is 3–20 higher in LMICs compared with high-income countries;[10] and expert opinion suggests that about 27% of these could be reduced with a clean delivery, whether at home or in health facilities.[11] Beyond childbirth, access to WATSAN in the home has broader implications for the health of newborns and mothers, and across the life cycle.[12]

A clean delivery requires: clean hands of the birth attendant, clean perineum, clean birth surface, clean cord preparation and cutting, and appropriate newborn postpartum skin care;[11] these ‘six cleans’ cannot be achieved without good access to WATSAN. Access to WATSAN in both the facility and home environment is generally very low across LMICs. A recent WHO report found that 38% of healthcare facilities across 54 countries did not have access to basic water sources and 19% to basic sanitation infrastructure.[13] The absence of water, sanitation and hygiene (WASH) services jeopardises birth attendants’ ability to carry out hygiene and relevant infection prevention and control practices, whether at home or in a facility. In 2015, 663 million people still lacked improved drinking water sources, and 2.4 billion people lacked improved sanitation facilities at home.[14] Hence, the new Sustainable Development Goals (SGD) recently reaffirmed access to WATSAN as a key global priority (SGD 6).[15]

While two recent studies describe the situation for WATSAN birth environment in Tanzania,[16,17] there is little research to understand the global reality. Even scarcer is information on how coverage of WATSAN at birth varies among and within countries. The Tanzania by Benova and colleagues found that women in the poorest quintiles bear a double burden: they are more likely to give birth at home and the proportion of home deliveries in a WATSAN-safe environment is at, or below 3% for all but the richest quintile. [16] The UNICEF Joint Monitoring Program (JMP) describes the status of home water and sanitation for the general population; however, the socio-economic distribution of women giving birth differs from the general population in that women giving birth are usually younger and poorer, and thus they are more likely to have worse water and sanitation than the general populations. Hence, it is important to investigate specifically the WATSAN home environment for births.

In this paper, we investigated the status of WATSAN in childbirth environments in low and middle-income countries to understand who delivers with access to basic WATSAN infrastructure. First, we described the home WATSAN environment among those who delivered at home by country, region, and women’s socio-demographic characteristics. We focused on world regions and countries where the proportion of home deliveries is higher. Second, we examined the WATSAN environment in health facilities in four countries in Eastern Africa: Kenya, Rwanda, Tanzania and Uganda, by facility type, delivery volume and managing authority; we chose these countries because Eastern Africa has substantial weaknesses in home and facility WATSAN and because of data availability. Third, for each of these four countries, we compiled home and facility results to describe what proportion of women delivered with access to basic WATSAN by country and by subnational region.

## Methods

To describe the water and sanitation status of home and facility childbirth environment, we relied on three distinct datasets created with publically available data: a global dataset, with information on the home environment among women who delivered at home from 58 countries, and two datasets for each of the four countries in Eastern Africa. These were the ‘healthcare facility’ dataset and the ‘Eastern Africa combined dataset’, which incorporates information on facilities, the home environment and a woman’s birth location to create a comprehensive description of birth environments in those countries. Where we used information on the home environment, we restricted the study sample to women who had had a live birth in their own household in the two years preceding the survey to allow for comparability between data sources. The childbirth experience represents each woman’s most recent birth.

### Global dataset

#### Data source and variables definition

To assess WATSAN in the home, we used publicly available datasets for LMICs from Demographic and Health Surveys (DHS)[18] and Multiple Indicator Cluster Surveys (MICS).[19] The dataset included 58 national surveys, the most recent available for each country carried out since 2000, with available information on the place of delivery, water source and sanitation infrastructure (Table A in S1 Table). We only analysed world regions with data from at least five countries where more than 100 women delivered at home; our intent was to produce estimates representative of those regions where the proportion of women delivering at home is substantial. We classified the five regions that fulfilled this criterion using the UNICEF regions: West and Central Africa, Eastern and Southern Africa, East Asia and Pacific, South Asia, Middle East and North Africa. We included 91%, 77%, 22%, 87%, 30% of countries from each region respectively (Table B, in S1 Table). Countries within each selected region with fewer than 100 women who delivered at home were excluded due to sample size concerns.

These datasets are generally representative of all women of reproductive age, usually 15–49 years for DHS and 15–44 for MICS–except those restricted to include only ever-married women. Both survey types contain detailed information about women’s most recent live birth. Our sample only includes women who had their most recent birth in their own home because only for those we could estimate their likely WATSAN environment at the time of delivery.

We constructed a variable to characterise the home WATSAN environment. An ‘improved WATSAN environment’ was one where both the drinking water source and sanitation access were improved in terms of infrastructure (improved water includes piped into dwelling, borehole etc.; improved sanitation includes flush toilet, septic tank etc.) according to the WHO/UNICEF Joint Monitoring Program (JMP) definition for households.[14] Examples of unimproved water infrastructure include an unprotected spring or dug well. Unimproved sanitation includes all sanitation infrastructures that are shared with other households, and infrastructure such as bucket or a pit latrine without a slab–even though not shared.

Socio-economic position was assessed using asset-based household indices, maternal education and rural/urban residence that were available from DHS and MICS datasets. Asset-based indices were derived using principal component analyses from variables representing household assets.[20] The first component was grouped into five quintiles (Qs) of households. Urban or rural residence was already defined in the datasets; this is done by MICSs/DHSs on a country basis, according to local census bureaus. Maternal education was classified as no education, any primary, and any secondary or higher. For Kenya, information on maternal education was not comparable to the other surveys (Table A in S1 Table); analysis with this variable was thus, not carried out for Kenya.

#### Analysis

Taking into account the sampling strategy using individual sample weights and clustering, we estimated the coverage of WATSAN among women who delivered at home for each country, each world region, and by three socio-demographic indicators: wealth index, maternal education and urban or rural residence. Regional values were estimated using the crude means and medians of all the countries in that region. Means and medians were not weighted by each country’s population size. To assess wealth-related inequalities in access to WATSAN we calculated the difference (absolute inequality) and the ratio (relative inequality) of Q5 (richest) and Q1 (poorest) WATSAN values.

### Eastern Africa

#### Data source and variable definitions—the healthcare dataset

WATSAN in healthcare facilities was investigated in Kenya, Tanzania, Uganda and Rwanda. These countries each had a recent Service Provision Assessment survey (SPA)–these are nationally representative surveys of health care facilities–[18] and a DHS in a similar timeframe (S2 Table). Moreover, they all belong to the Eastern and Southern Africa region, which has substantial weaknesses in home and facility WATSAN [13,14]. We based our analysis on a restricted sample of facilities providing routine delivery services.

The categorisation of facility types differed between countries. Using the SPA country reports we examined facility levels and functions across the countries to classify facilities into three main categories: hospitals, health centres and dispensaries (S3 Table); and by managing authority (private or public).

**Fig 1:**
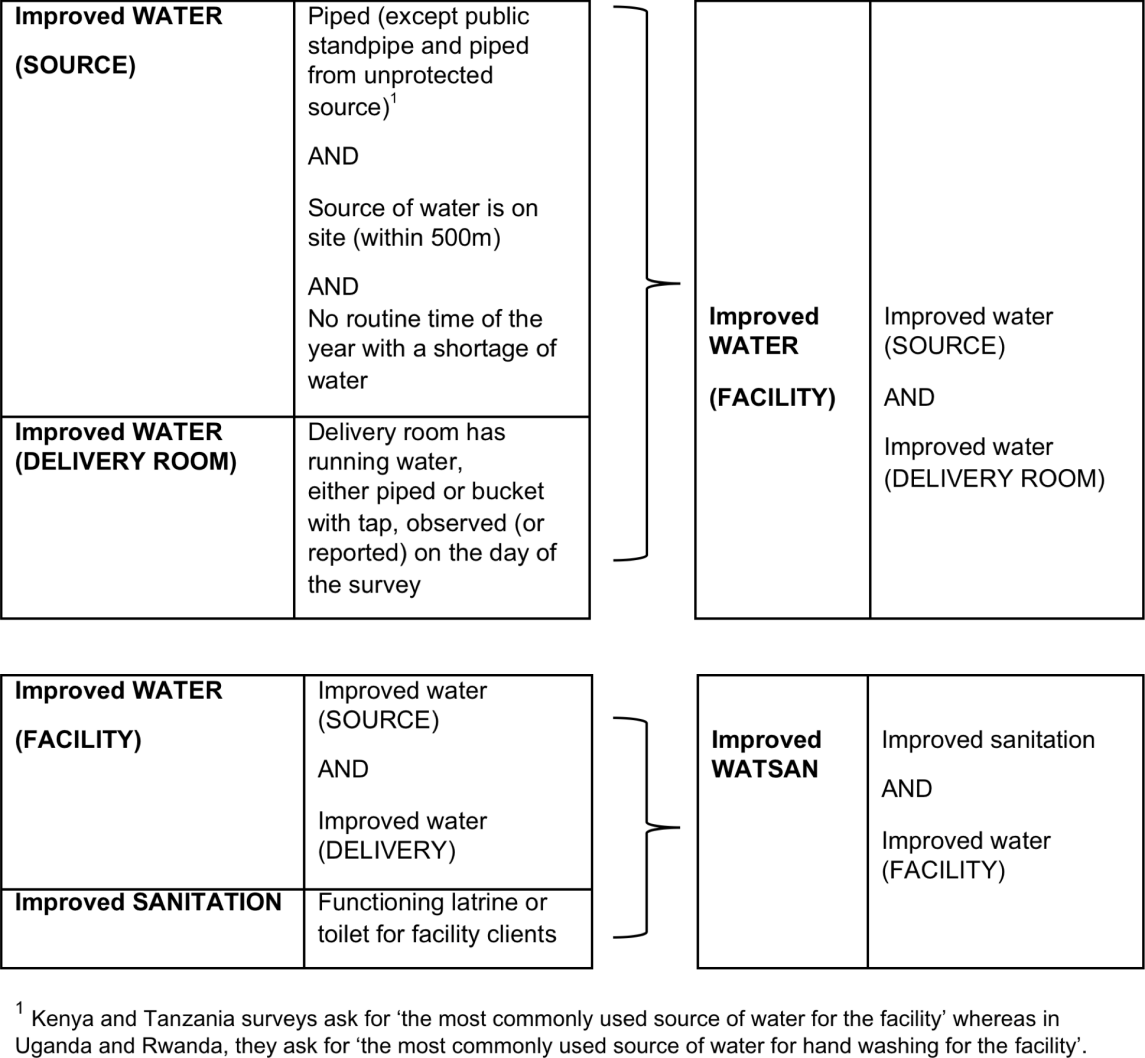
Facility WATER and WATSAN indices.

We created two main indices, which differed from the components used in the home index. The first was for improved WATER in the facility, which includes a measure for improved water source in the facility and running water–either piped or bucket with tap–in the delivery room; the second was for improved WATSAN, which includes the WATER index and information on sanitation (Fig 1).

The facility WATER index required stricter criteria for water than the home index because healthcare facilities can receive very large volumes of deliveries and thus water needs to be constantly available at the point of care. In addition, water is also more vital for environmental cleaning in a setting where the volume of ill patients increases the risk of contamination. There is no international standard definition for water, for sanitation or their combination for health facilities. We based our indices on the WHO report[13] and the classification proposed by Benova et al [16] with a slight modification explained in the next paragraph, and added a criterion of having a continuous water supply (no time of the year with a routine shortage of water), a necessary condition for improved WATER, and hence WATSAN. At community level, the criterion of continuous supply is effective in reducing diarrhoeal disease [21]–plausibly because it allows people to use water for infection prevention behaviours, such as washing hands, environmental cleaning, and the higher quality and quantity of drinking-water. These behaviours are also fundamental during labour and delivery for maternal and newborn sepsis prevention, and justify the additional criterion.

The slight modification was that we reasoned that the main water source should be exclusive to the facility to avoid delays in access. Therefore unlike the JMP, WHO and Benova et al definitions, we considered those facilities where the water source was a ‘public tap/standpipe’ (Rwanda: 8%, Kenya: 1%, Uganda: 1%) to be ‘unimproved’. Also, we considered ‘piped water’ as ‘improved’, if it was from an ‘unknown’ source, because the information on the water source was provided by a healthcare worker who may have had little knowledge on this; it was classified as ‘unimproved’ if the respondent specifically chose the option ‘piped from an unprotected source’ (Tanzania: 3%).

The criteria for SANITATION we used also differed from the home criteria. No country collected information about latrines/toilets located in the maternity, which would have been our ideal measure, especially in larger facilities. In Kenya and Tanzania, we used information about the availability of functional general facility latrines/toilets; functionality was not available for the home SANITATION definition. Information on the type of toilet in facilities, used in the JMP definition for home SANITATION was not available for facilities. We only examined WATER in Uganda and Rwanda because they did not have sanitation data for three-quarters of facilities performing deliveries in Rwanda and one-quarter in Uganda.

#### Data source and variable definitions—the combined dataset

We used data from four DHS surveys, restricting the sample to women who delivered either in their own home or in a facility of a known type. Those who delivered in *other* locations (4% to 10% across the four countries) were not included because we did not have information on their likely WATSAN environment at the time of delivery (see details on this *other* category in S4 Table).

To allow for comparability between SPA and DHS, we created a ‘place of delivery’ variable (described in S4 Table). We used the same variable for improved WATER and WATSAN in the home as that described for the global dataset. Women in the DHS who delivered in their own home were allocated home WATSAN (Tanzania and Kenya) or WATER (Uganda and Rwanda) values. Women in the DHS who delivered in a facility were allocated the average of improved facility WATER or WATSAN for their region, calculated from the SPA within the “healthcare dataset” (details of this method in S1 File). Previous work on linking DHS and SPA datasets without using GPS coordinates suggested linking the two at a level at which the surveys were representative; hence we used this method too.[22] For Tanzania and Uganda, we recoded regions to allow comparability between the SPA and DHS (S5 Table).

#### Analysis—Eastern Africa

When analysing both datasets we accounted for the sampling frame (sample weights, clustering and stratification) using the *svyset* commands. In addition, for the SPA analysis only, we created an additional set of weights—*delivery (volume) weights;* these accounted for the proportion of deliveries carried out by each facility compared with the total number of facility deliveries for that country (S2 File). Our intention was to present the proportion of facility deliveries that occur in an improved WATER or WATSAN environment at country level. Information on the number of deliveries, used to produce the weighting by number of delivery was missing to a different extent in in each country, but never exceeded 8%. We ran complete case series analyses.

Using the healthcare dataset, we carried out descriptive statistics to calculate the proportion of facilities or facility deliveries with improved WATSAN or WATER, by country, facility type and managing authority. Using the Eastern Africa dataset, we estimated what proportion of women delivered with improved WATER and improved WATSAN, nationally and by subnational region. We analysed all three datasets in Stata/SEv.14, using publicly available data.

### Ethical procedures and approvals

For both the SPAs and the DHSs, the Institutional Review Board (IRB) of the country where the survey takes place ensures that the survey complies with the country regulations. Whereas, ICF International IRB ensures it complies with the U.S. Department of Health and Human Services regulations for the protection of human subjects. For more information please refer to: http://dhsprogram.com/What-We-Do/Protecting-the-Privacy-of-DHS-Survey-Respondents.cfm.

For the DHS surveys, typically the written informed consent is read by the interviewer and includes the purpose of the study, that the participation is voluntary and data would be confidential and anonymised. The respondent can decline or accept verbally to consent and this is recorded on the survey tool using the interviewer signature.

Ethics for the MICS surveys is responsibility of the body and country who conducts it. Guidance for conducting MICS survey suggests that the survey must abide the laws of the country and apply for local ethics approval, that all information should be confidential, that respondents should given their full approval to the request of consent verbally unless written consent is required by the country where the survey takes place. In addition, useful feedback is expected to be given to participants and their community; for example, mothers should be advised when their children’s vaccinations are overdue.

Across the four SPAs for Kenya, Tanzania, Uganda, and Rwanda, informed consent was verbally obtained from the facility in-charge, and from all respondents for the facility, and recorded by the interviewer on the survey tool using the interviewer signature. Consent from respondents involved explaining them about the purpose of the study, that no patient names would be reviewed, recorded or shared, that they might refuse to answer any question and that they can stop the interview at any time, and that facility names would be anonymised. Respondents were also told that the information about their facility may be used by their Ministry of Health or organizations supporting the facility, or researchers for planning service improvement or further studies of health services.

Our secondary analyses of these anonymised datasets were approved by the Observational/Interventions Research Ethics Committee at the London School of Hygiene and Tropical Medicine. The sources of data are available for DHSs and SPAs at www.measuredhs.com and for MICSs at http://mics.unicef.org/.

## Results

### Global Analysis

The sample size of women delivering at home, weighted by the sample characteristics, is available for each country is in S6 Table and ranges from 101 to 28979. The proportion of missing responses for home WATSAN was less than 2% across all 58 surveys. Table 1 shows that the average proportion of women delivering at home varies greatly by region, with the highest being in East Asia and Pacific (53%) and the lowest in Middle East and North Africa (28%).

**Table 1:** Mean and median proportion of women delivering at home and improved home WATSAN among women who delivered at home, by world region *(DHS and MICS data)*.

World region	Number of countries	Proportion of women who delivered at home		Coverage of improved WATSAN among women who delivered at home	
		Mean	Median	Mean	Median
Eastern & Southern Africa	17	32.6	32.1	13.3	9.3
West & Central Africa	22	33.9	30.8	9.1	6.7
Middle East & North Africa	6	28.3	26.3	52.5	52.6
South Asia	7	48.4	51.4	34.0	27.3
East Asia & Pacific	6	53.0	54.4	24.0	24.8

Among women who delivered at home, regional coverage of improved WATSAN in the home varied between 9% in West and Central Africa to 53% in Middle East and North Africa (Table 1). Within regions, variation was also striking–for example within the Middle East and North Africa, the mean improved WATSAN in Sudan was 14%, whereas in Egypt it was 87% (S6 Table). Improved WATSAN coverage by country is given in S6 Table.

**Fig 2:**
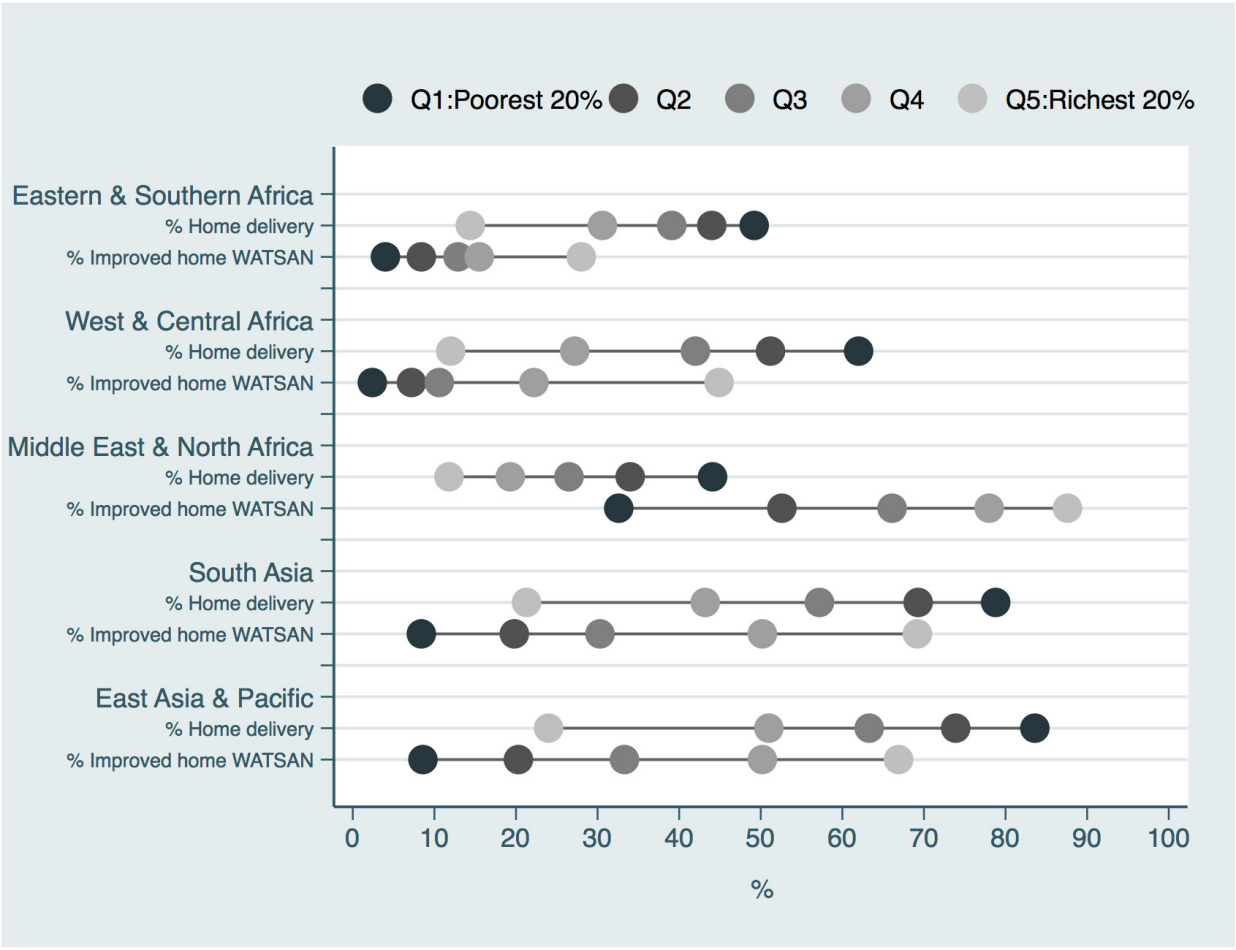
Proportion of home births and coverage of improved WATSAN among women who delivered at home, by wealth quintile and world region *(DHS and MICS data)*.

Fig 2 shows the coverage of home improved WATSAN for wealth quintiles by region. We observed a monotonic pattern in the coverage of improved WATSAN that increased with higher wealth quintiles across all the regions investigated. Eastern and Southern Africa, and West and Central Africa showed the lowest coverage (less than 50%) of improved home WATSAN across all quintiles. Middle East and North Africa, and West and Central Africa showed substantial inequalities: in the former, the poorest lagged behind; in the latter the richest were substantially better off. Distribution of improved WATSAN coverage by education and rural/urban area respectively produced similar findings to those stratified by wealth index (S1 and S2 Figs).

In terms of absolute inequality, calculated as the difference in percentage points (pp) between the women in the richest and poorest quintiles for improved home WATSAN, was lowest in Eastern and Southern Africa (24pp) (Table 2). Higher absolute inequality was seen in South Asia and the Pacific, in the Middle East and North Africa and in East Asia and Pacific (respectively at 61pp, 55pp and 58pp). Fig 2 shows absolute inequalities visually; longer lines between Q1 (poorest) and Q5 (richest) represent larger absolute inequalities. In terms of relative inequality, calculated as the ratio of improved home WATSAN between the richest and poorest quintiles, it was lower (i.e. lower relative inequality) in Middle East and North Africa with 2.7. The highest ratio of 18.5 was in West and Central Africa (Table 2).

**Table 2:** Mean and median coverage of improved WATSAN by wealth quintile, and absolute and relative inequalities between the lowest and the highest wealth quintile by world region (*DHS and MICS data)*.

World region	Wealth quintile	Proportion of women with home improved WATSAN	Absolute inequality	Relative inequality
			(Q5-Q1)	(Q5/Q1)
Eastern & Southern Africa	Poorest	4.0		
	Poorer	8.4	24.0	7.0
	Middle	12.9		
	Richer	15.5		
	Richest	28.0		
West & Central Africa	Poorest	2.4		
	Poorer	7.2	42.5	18.5
	Middle	10.6		
	Richer	22.2		
	Richest	44.9		
Middle East and North Africa	Poorest	32.6		
	Poorer	52.6	55.0	2.7
	Middle	66.1		
	Richer	78.0		
	Richest	87.6		
South Asia	Poorest	8.4		
	Poorer	19.8	60.8	8.2
	Middle	30.3		
	Richer	50.2		
	Richest	69.2		
East Asia & Pacific	Poorest	8.6		
	Poorer	20.3	58.3	7.7
	Middle	33.3		
	Richer	50.2		
	Richest	66.9		

Fig 2 shows, especially in the poorer regions (Eastern and Southern Africa, West and Central Africa, South Asia, and East Asia and Pacific), that while coverage of WATSAN increases with increasing wealth, for home deliveries, the sequence of dots is reversed as the proportion of home deliveries decreases with wealth. This is what we refer to as the double burden of poverty; poorer women are more likely to deliver at home and have worse WATSAN compared to their richer counterparts. In West and Central Africa, those in the poorest quintile were five times more likely to deliver in their own home, and were 18 times less likely to have improved home WATSAN coverage. The double burden of poverty was less striking in the wealthier Middle East and North Africa.

### Eastern Africa: Kenya, Tanzania, Uganda, Rwanda

Overall in the four countries investigated, the percentage of missing data was low (up to 6%) for the SPA datasets and even lower in the DHS (0.1% or less) (Table 3). All results presented were weighted by the sample weights provided in the datasets unless specified.

**Table 3:** Distribution of births by place of delivery and by country *(DHS data)*.

Place of delivery	KENYA	TANZANIA	UGANDA	RWANDA
	% (CI)	% (CI)	% (CI)	% (CI)
**Own home**	51.5% (47.4%-55.5%)	45.4% (42.3%-48.5%)	33.7% (30.7%-36.8%)	19.6% (17.9%-21.5%)
**Hospital**	24.4% (21.9%-27.2%)	28.0% (25.6%-30.6%)	19.0% (16.9%-21.3%)	18.6% (17.1%-20.3%)
**Health centres**	8.3% (6.7%-10.3%)	9.4% (7.9%-11.2%)	32.2% (29.5%-35.0%)	60.8% (58.7%-62.8%)
**Dispensaries**	3.2% (2.3%-4.4%)	17.1% (14.9%-19.5%)		0.9% (0.6%-1.4%)
**Private facilities**	6.9% (5.6%-8.4%)		15.1% (13.0%-17.3%)	
**Mission facilities**	5.7% (4.4%-7.3%)			

From Table 3, about half of the women delivered their most recent birth in the home in Tanzania (45%) and Kenya (52%) compared with a third in Uganda (34%,) and 20% in Rwanda. In Tanzania and Kenya, about a quarter of women delivered in hospitals, a higher proportion than in Uganda and Rwanda. Less than 10% of women delivered in health centres in Kenya and Tanzania, but the proportion is higher in Uganda (32%) and Rwanda (61%). With the exception of Tanzania at 17%, 3% or fewer women delivered in dispensaries.

**Fig 3:**
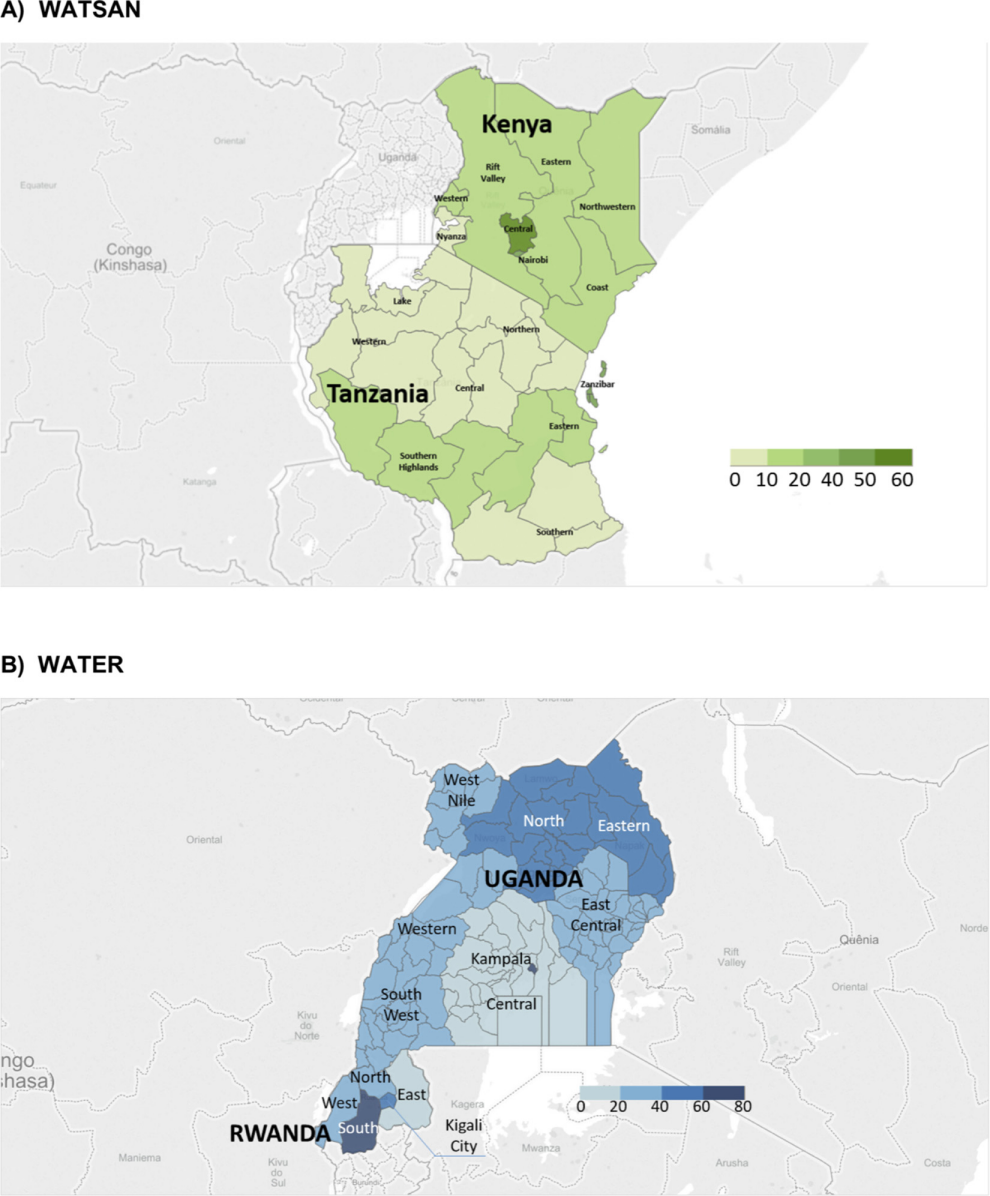
Proportion of improved WATER and WATSAN among women who delivery in either a facility or at home, by country and region *(SPA and DHS data)*.

Fig 3 shows the coverage of improved WATSAN (A) and WATER (B) for the childbirth environment, combining information for home and healthcare facility deliveries. In most regions in Uganda and Rwanda, between 20% and 40% of women delivered in an improved WATER environment. There appeared to be more regional variation in Uganda (11% in the Central region and 75% in Kampala) compared with Rwanda. Improved WATSAN for the childbirth environment fell within the range 10–20% in most regions across Kenya and Tanzania. There was higher regional variation in Kenya (6% in Nyanza, and 51% in Central region) compared with Tanzania. Nationally, about 30% of women delivered in an environment with improved WATER in Uganda (33%) and Rwanda (30%); whereas, 18% of women in Kenya and 7% in Tanzania delivered with improved WATSAN.

The unweighted proportion of facilities providing normal delivery services in each country was 30% (207 facilities) in Kenya, 74% (454) in Tanzania, 54% (265) in Uganda, and 76% (407) in Rwanda respectively (S3 File).

**Fig 4:**
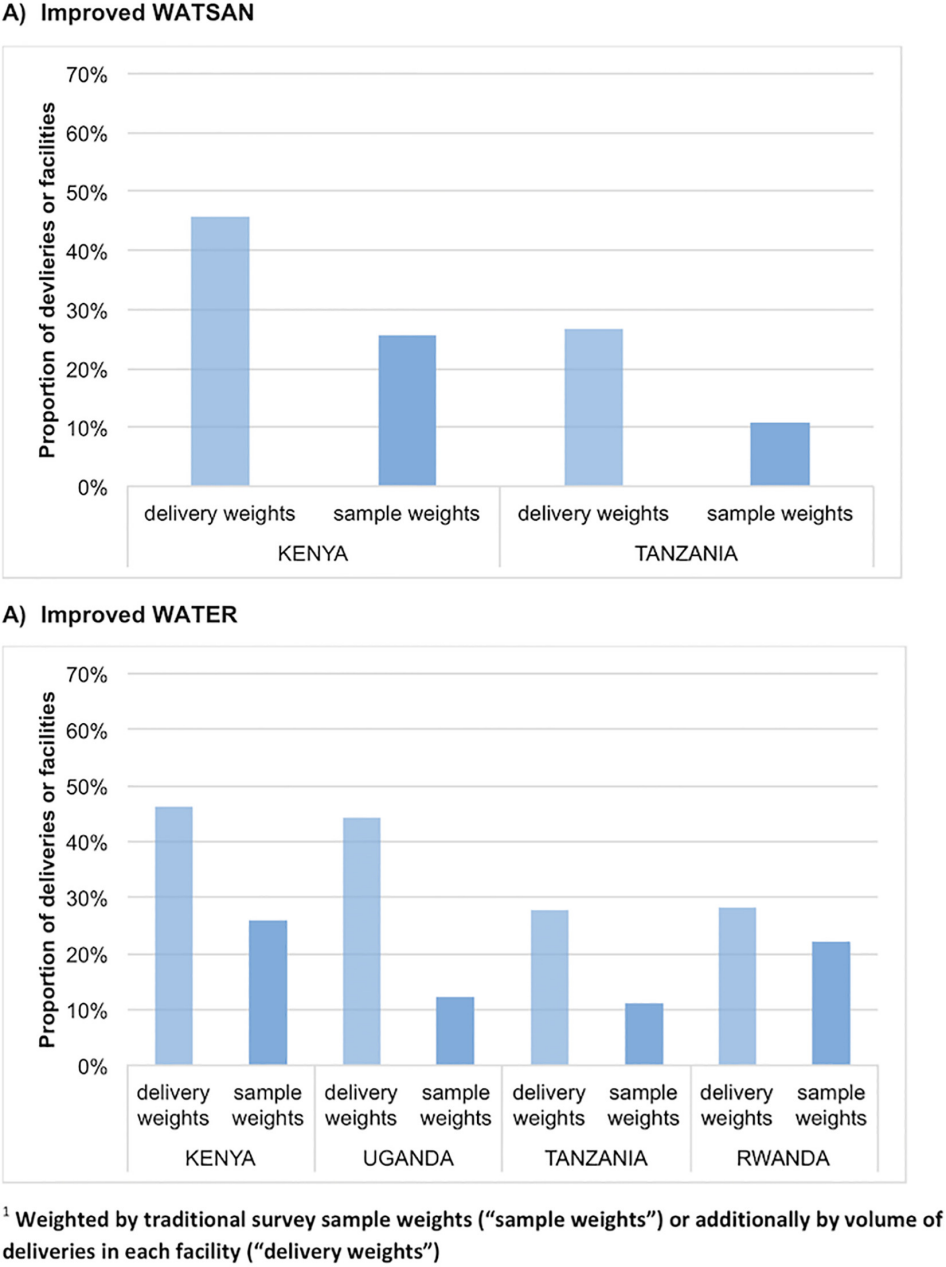
Proportion of facilities or facility deliveries with improved WATSAN or improved WATER, by country and by weighting methods (weighted by traditional survey sample weights “sample weights”, or additionally by volume of deliveries in each facility “delivery weights”)^1^. (SPA data).

The proportion of facilities with improved WATSAN (A) and WATER (B) was below 30% for all countries (Fig 4). Yet when weighted by the volume of facility deliveries, coverage appears higher, although still below 50%, for both improved WATSAN (A) and WATER (B) across all countries. This was because more deliveries occurred in higher-level facilities where there was better WATSAN. Results in Fig 4, we restricted the analysis to those facilities that have information on delivery number to ensure comparability between those weighted by the number of deliveries and those using the standard sample weights.

Overall, over 90% of facilities in Tanzania (90%) and Kenya (99%) had improved SANITATION, so the WATSAN index could mostly be explained by the lack of improved WATER at facility level. For all four countries, improved WATER coverage was brought down by WATER source indicators, rather than the delivery room indicator (S7 Table). Across the countries about half of facilities experienced water shortages and everywhere, expect Rwanda at 37%, a similar or higher proportion did not have a piped water supply. Having a water source further than 500m from a facility was more common in Rwanda (27%) and Tanzania (40%).

**Fig 5:**
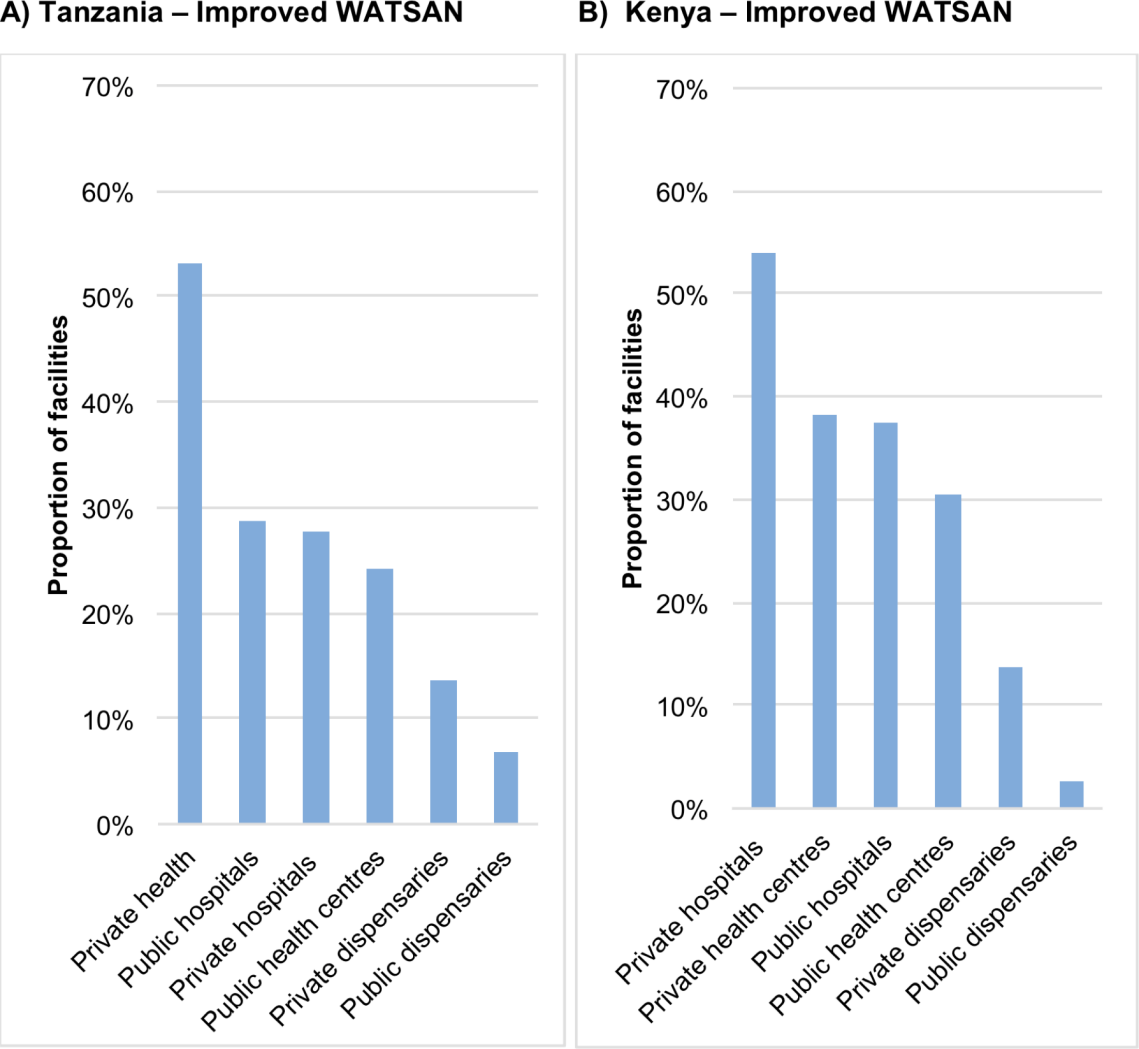
Proportion of facilities with improved WATSAN by facility type and managing authority A) Tanzania and B) Kenya, using sample weights. *(SPA data)*.

**Fig 6:**
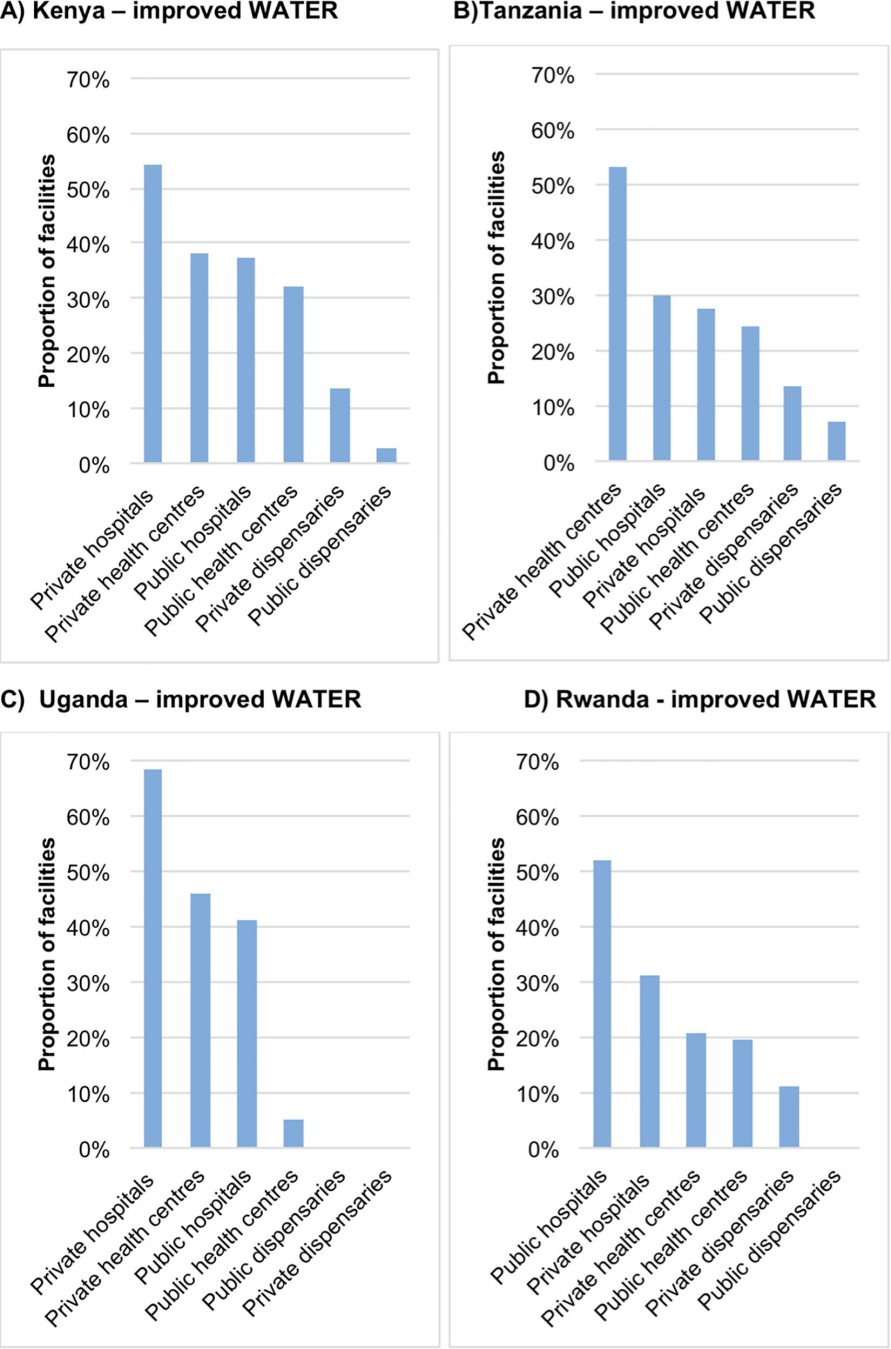
Proportion of facilities with improved WATER by facility type and managing authority: A) Kenya and B) Tanzania, C) Uganda and D) Rwanda, using sample weights. *(SPA data)*.

Fig 5 shows private facilities held the highest proportion, just above 50%, of improved facility WATSAN in both Tanzania (health centres) and Kenya (hospitals). Those with the lowest proportions were public dispensaries (below 10%) followed by private dispensaries (below 20%). The pattern of results was similar for improved facility WATER–with the exception of Rwanda were public hospitals score the highest coverage of improved WATER (Fig 6).

## Discussion

The descriptive analyses of the three cross-sectional datasets shows that women who delivered at home, particularly in Sub-Saharan Africa, had poor access to WATSAN infrastructure and that this was worse among the poorest, the less educated and those living in rural areas. In Eastern Africa, both home and facility childbirth environments had very poor access to WATER or WATSAN.

As far as we are aware, our results are the first attempt to describe the WATSAN status of childbirth environments across low and middle-income countries, in both facility and home. We used 58 nationally representative surveys for the home analysis, covering five of the UNICEF world regions. Our results are representative of countries in these regions with at least 100 women delivering at home. Across countries in both the global and Eastern Africa datasets, our results are representative of 90% of deliveries–including women who delivered in their own home or in a facility of a known type.

Consistent with similar analyses describing the home WATSAN environment across the world, [14] we found that West and Central Africa, and Eastern and Southern Africa had the lowest coverage of improved home WATSAN, less than 15%). The regional estimates we present are generally lower than those presented by the UNICEF/JMP for the general population; this is most likely to be explained by the socio-economic distribution of women giving birth at home differing from the general population by being younger and poorer. As reported for other coverage indicators in other studies, for virtually every region, we observed a monotonic pattern in the coverage of improved home WATSAN with higher coverage among those women in the richer quintiles, having higher levels of education and living in urban areas.[23, 24] Although the number of countries per world region can be small, we chose to present the results of the global analysis using means rather than median–although both are in our table (Table 1). They yielded similar estimates, but the interpretation of means tends to be accessible to a wider audience, compared to medians.

Relative inequalities in wealth–the degree of disparity between the poorest and richest quintile–were highest in West and Central Africa. Consistent with an analogous analysis for Tanzania, [16] we observed that poorer women tended to deliver at home and have worse home WATSAN across all regions compared with those in richer quintiles. This double burden of poverty (i.e. associated with both more home deliveries and worse WATSAN conditions) was more evident across the lowest-income regions: West and Central Africa, Eastern and Southern Africa, South Asia, and East Asia and Pacific. The wealth asset-based index we used to investigate socio-economic differentials was available in the dataset and included water and sanitation variables. We do not believe this biased the results because most country wealth indices included over 30 other assets.[25]

A more in-depth analysis of Eastern Africa, allowed us to investigate WATSAN coverage in both home and facility environments. Overall, about a third of women in Uganda and Rwanda delivered in an environment with improved WATER; whereas, 18% of women in Kenya and 7% in Tanzania delivered with improved WATSAN. From our analysis we found that within each country there was substantial regional variation; this is consistent with similar work on the topic.[13,16]

To estimate regional coverage of improved WATSAN and WATER across the four countries we linked SPA and DHS surveys, initially not designed for this purpose, at the level, the region, at which they were both representative, as recommended by others.[22] We are confident in our results because we tried two distinct methods to obtain them and both yielded similar findings.

Across healthcare facilities providing maternity services in the Eastern Africa dataset, coverage of WATER or WATSAN was below 30%, similar but lower than the 41% described by the WHO report for five countries using SPA (i.e. Haiti, Kenya, Namibia, Rwanda and Tanzania). Unlike the WHO WATER indicator, our indicator also included whether the delivery room had running water (either piped water, or a bucket with a tap).[13] An internationally agreed indicator to monitor access to WATSAN in maternity rooms does not exist yet[13]; the rationale for our proposed definition is detailed in the methods and should be considered when interpreting our results. When we accounted for the volume of deliveries occurring in each facility, the picture was more positive because higher-level facilities, such as health centres and hospitals, with the highest volume of deliveries, and had better WATSAN infrastructure. Private facilities, mostly hospitals, had the highest proportion of improved facility WATER and WATSAN coverage (above 50%). Only Rwanda had the highest WATSAN coverage amongst public hospitals. This may be related to the government’s recent focus to provide higher and equitable access to delivery services.[26,27] The lowest coverage, as expected, was among public dispensaries, followed by private dispensaries. Yet results for these different levels of facility types should be interpreted with caution. Classification varies greatly between countries and it is plausible that some dispensaries in one country might provide similar services to a health centre in another; likewise, a health centre in one country might be considered a hospital in another. We relied on individual countries’ classifications for this analysis. In addition, governments are less likely to have accurate information on private facilities, especially smaller ones, than on public ones. Smaller and less well-known facilities are likely to have to have worse WATSAN; hence it is likely that the picture for private facilities is better than the reality. This might bias the results against public facilities.

Our analyses attempted to unpack those elements of the WATER and WATSAN indices’ components that contributed more substantially to low coverage across the four East African countries. Most of the low WATSAN coverage was explained by the lack of improved WATER, compared with SANITATION. This should be interpreted in the light of the fact that we postulated four different conditions for the water index to be met; while prescribing only one condition for SANITATION. Among the WATER index components, the most frequently not met were not having access to piped water and experiencing routine seasonal water shortages. Motivation of managers, at the hospital and ministry levels, to fix such issues timely, is a fundamental part to solving water shortage; however currently the SPAs do not include this information.

Our analyses rely on the assumption that women who delivered in their own home had a similar level of WATSAN infrastructure in their home at the time of delivery to that they reported when interviewed. By restricting our analyses to births in the two years prior to the survey, we believe that misclassification of improved WATSAN from this was minimal. Another assumption was that general environment latrines/toilets in healthcare facilities in Kenya and Tanzania are accessible to women in the maternity areas. Ideally we would have information on latrines/toilets specific to the maternity area, but this information was not available.

Misclassification of WATSAN had the greatest scope to limit our results. We have assumed that respondents of both the household and facility surveys were able to report information on their WATSAN type accurately. This was an issue particularly for the question around the water source in healthcare facilities, on which an average healthcare worker might report. To minimize potential bias, we considered water improved if piped from either an improved or unknown source. Another cause of uncertainty was whether respondents interpreted the question on water source as the water type at the original source, or when it reaches the facility–for example, if dug well water was piped into the facility grounds, then it is unclear what the appropriate response would be. Limited by data availability in the datasets, we only had information on the type of infrastructure, access and reliability, not on *cleanliness*. Ideally we would have had information on whether the water stored in bucket had a lid, on microbiological data on the quality of the water.[28] Information on whether the household or facility performed water filtering and treatment would also be important.[21] With regards to sanitation, ideally we would have information on the type of toilet or latrine available in the facility, access to toilets in the maternity and the cleanliness of the toilets. Finally, because additional necessary items to perform hygiene at birth, e.g. soap, were available in the SPAs but not consistently in all DHSs and MICSs, we decided not to measure availability of soap in these analyses. Ideally, however, all future DHS/MICS surveys would capture this information.

Too many women across the world and in particular in West and Central Africa, and Eastern and Southern Africa deliver at home without access to basic WATSAN. This has major implications for maternal and newborn health and survival. Inequality of access was striking across and within countries. Within the Eastern African region, we found that even among facility deliveries, less than half were in a childbirth environment with access to basic WATSAN. Access to WATSAN during childbirth should be routinely monitored in facilities across more countries. An agreed definition of WATSAN in maternities would enhance standardised monitoring, just as the JMP did for home WATSAN.[13,14] Targeted investments in facilities can guarantee essential resources for practicing infection prevention during childbirth, ensure an enabling environment for hygiene and ultimately reduce healthcare associated infections. [13,29–31]

## Supporting Information

S1 Fig Mean coverage of WATSAN among those who delivered at home, by maternal education and world region. (TIFF)

S2 Fig Mean coverage of WATSAN among those who delivered at home, by rural/urban and world region. (TIFF)

S1 File Linking SPA and DHS. (PDF)

S2 File Delivery weights. (PDF)

S3 File Distribution of facilities by type and country. (PDF)

S1 Table Data Availability: A) data availability according to country (most recent survey since 2000) and B) number of countries included in the analyses according to world region.
(PDF)

S2 Table Data sources for Eastern Africa analysis. (PDF)

S3 Table Facility type classification. (PDF)

S4 Table Classification of the place of delivery variable for the Eastern African dataset (PDF)

S5 Table Regions re-classification. (PDF)

S6 Table Improved home water, sanitation and WATSAN among women who delivered at home by country. (PDF)

S7 Table Unpacking the WATER index: percentage of facilities (calculated using sample weights) with or without each of the binary WATER index components. (PDF)
