# Supplementary figures and images for: Who Delivers without Water? A Multi Country Analysis of Water and Sanitation in the Childbirth Environment
Source: PLoS One. 2016 Aug 17;11(8):e0160572. doi: 10.1371/journal.pone.0160572 (PMC4988668; doi:10.1371/journal.pone.0160572)

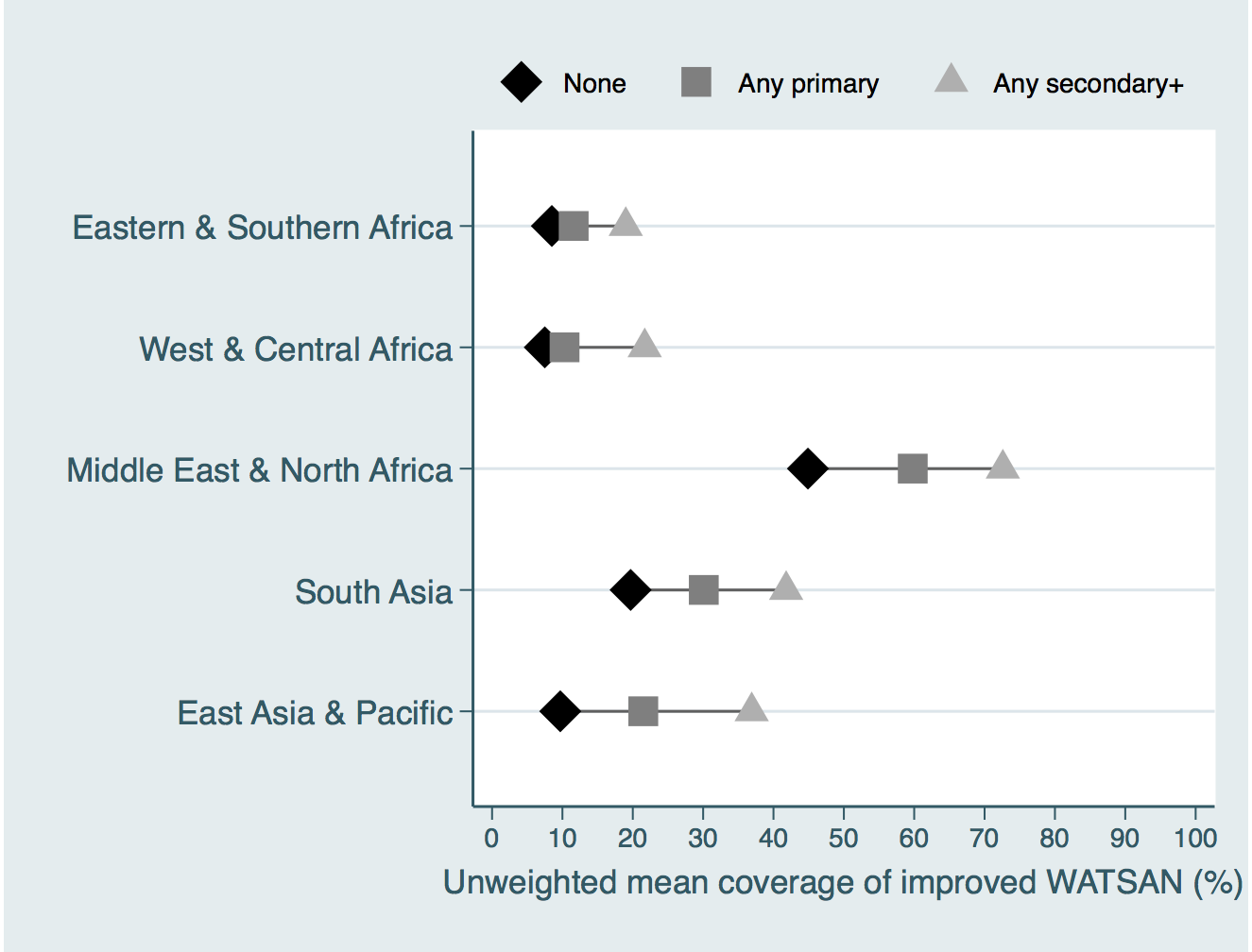

Supplement: S1 Fig — (TIFF) [file pone.0160572.s001.tiff]

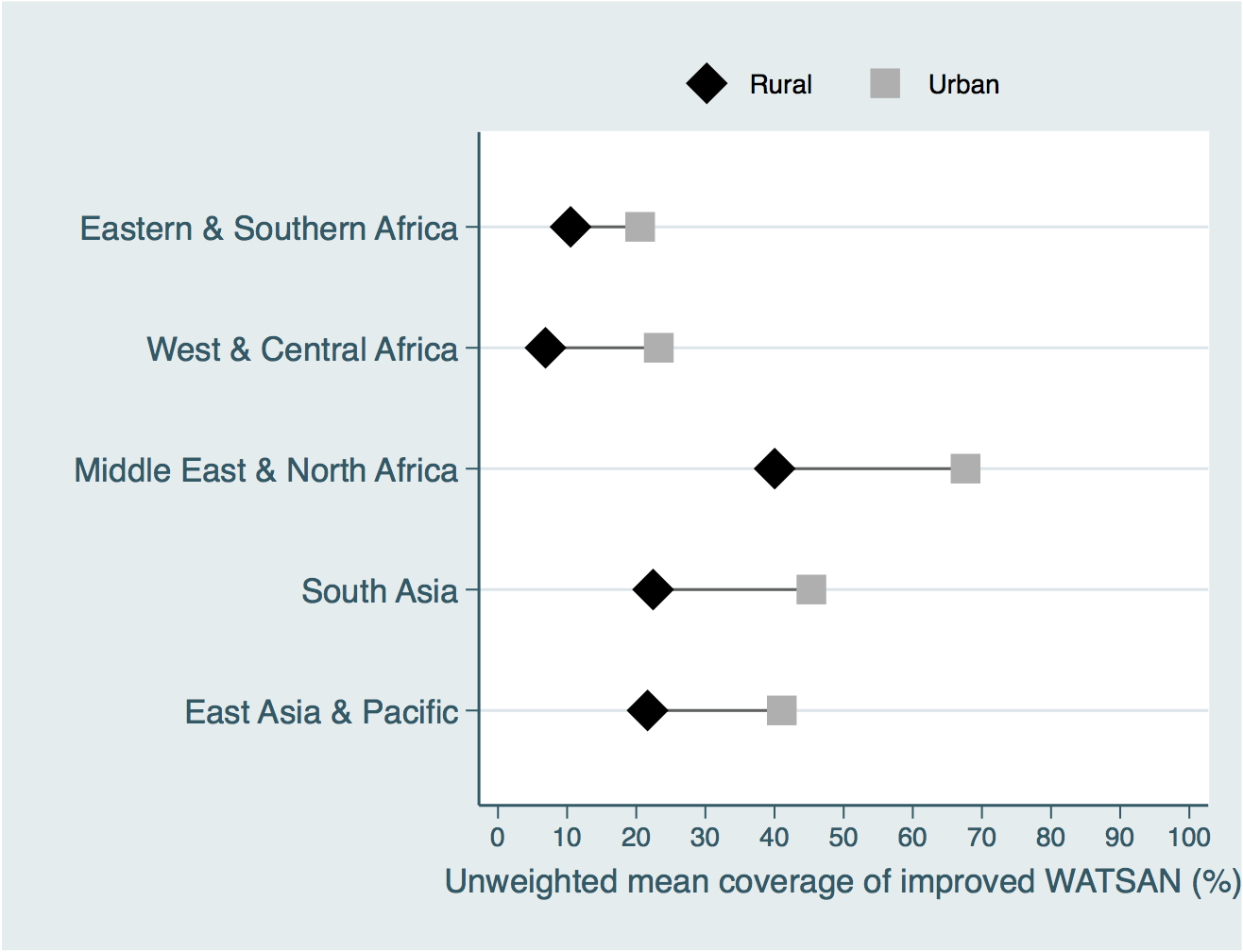

Supplement: S2 Fig — (TIFF) [file pone.0160572.s002.tiff]
