# Supplementary material for: Who Delivers without Water? A Multi Country Analysis of Water and Sanitation in the Childbirth Environment
Source: PLoS One. 2016 Aug 17;11(8):e0160572. doi: 10.1371/journal.pone.0160572 (PMC4988668; doi:10.1371/journal.pone.0160572)
Supplement: S1 File — (PDF) [file pone.0160572.s003.pdf]

The *regional* average was chosen over an average estimated by taking the average of each facility type within a specific region (*region&facility type* method) because: a) within each facility type by region, facility count was low in some regions; b) previous work on linking DHS and SPA datasets without using GPS coordinates suggest linking the two at level at which the survey is representative; c) we calculated the proportion of improved WATSAN or WATER by country regions using both methods (*region&facility type and regional* methods*)* and yielded very similar results. Across 29 regions in the four countries, the two methods provided estimates falling within the same 10% range for 25 of them. For 3 regions the *regional* method was yielding slightly lower estimates that fell in the lower 10% range compared to the *region&facility type* method. For only one region in Rwanda the difference between the two methods yielded considerably different estimates. Finally, we decided to use the *region&facility type* method also because the Rwanda DHS does not have a response category for having delivered in a private hospital; whereas, from the Rwanda SPA, we know 35% of facility deliveries occur in private facilities. Using the weighted estimates by region, we bypassed this inconsistency between the two datasets.
