# Supplementary material for: Who Delivers without Water? A Multi Country Analysis of Water and Sanitation in the Childbirth Environment
Source: PLoS One. 2016 Aug 17;11(8):e0160572. doi: 10.1371/journal.pone.0160572 (PMC4988668; doi:10.1371/journal.pone.0160572)
Supplement: S2 File — (PDF) [file pone.0160572.s004.pdf]

To create the *delivery weights*, the standard survey sample weights provided in the SPA dataset were multiplied by the number of deliveries in the previous 12 months; this was then divided by the sum of all deliveries in the previous 12 months in that country.
