# Supplementary material for: Who Delivers without Water? A Multi Country Analysis of Water and Sanitation in the Childbirth Environment
Source: PLoS One. 2016 Aug 17;11(8):e0160572. doi: 10.1371/journal.pone.0160572 (PMC4988668; doi:10.1371/journal.pone.0160572)
Supplement: S3 File — (PDF) [file pone.0160572.s005.pdf]

Each country included the following number of facilities providing normal delivery services by type (unweighted):

- Kenya: 207 facilities overall of which 30 are hospitals, 49 public health centres, 46 are public dispensaries, 45 are private facilities and 37 are mission facilities.
- Tanzania: 454 facilities overall of which 24 are hospitals, 48 health centres, 382 are dispensaries.
- Uganda: 265 facilities overall of which 10 are public hospitals, 190 are government health centres, and 64 are private facilities.
- Rwanda: 407 facilities of which 39 are hospitals, 348 health centres and 20 are dispensaries.
