# Supplementary material for: Who Delivers without Water? A Multi Country Analysis of Water and Sanitation in the Childbirth Environment
Source: PLoS One. 2016 Aug 17;11(8):e0160572. doi: 10.1371/journal.pone.0160572 (PMC4988668; doi:10.1371/journal.pone.0160572)
Supplement: S1 Table — Data Availability: A) data availability according to country (most recent survey since 2000) and B) number of countries included in the analyses according to world region. (PDF) [file pone.0160572.s006.pdf]

A)

Country	Survey year	Type of survey	Place of delivery	Improved safe water	Improved sanitation	Wealth index (quintiles)	Area	Maternal education
Afghanistan	2010	MICS	✓	✓	✓	✓	✓	✓
Bangladesh	2011	DHS	✓	✓	✓	✓	✓	✓
Benin	2011	DHS	✓	✓	✓	✓	✓	✓
Bhutan	2010	MICS	✓	✓	✓	✓	✓	✓
Burkina Faso	2010	DHS	✓	✓	✓	✓	✓	✓
Burundi	2010	DHS	✓	✓	✓	✓	✓	✓
CAR	2010	MICS	✓	✓	✓	✓	✓	✓
Cambodia	2010*	DHS	✓	✠	✓	✓	✓	✓
Cameroon	2011	DHS	✓	✓	✓	✓	✓	✓
Chad	2010	MICS	✓	✓	✓	✓	✓	✓
Comoros	2012	DHS	✓	✓	✓	✓	✓	✓
Congo Brazz	2011	DHS	✓	✓	✓	✓	✓	✓
Congo DR	2013	DHS	✓	✓	✓	✓	✓	✓
Cote d'Ivoire	2011	DHS	✓	✓	✓	✓	✓	✓
Djibouti***	2006	MICS	✓	✓	✓	✗	✓	✓
Egypt	2008	DHS	✓	✓	✓	✓	✓	✓
Ethiopia	2011	DHS	✓	✓	✓	✓	✓	✓
Gabon	2012	DHS	✓	✓	✓	✓	✓	✓
Gambia	2013	DHS	✓	✓	✓	✓	✓	✓
Ghana	2011	MICS	✓	✓	✓	✓	✓	✓
Guinea	2012	DHS	✓	✓	✓	✓	✓	✓
Guinea Bissau	2006	MICS	✓	✓	✓	✓	✓	✓
India	2005	DHS	✓	✓	✓	✓	✓	✓
Indonesia	2012*	DHS	✓	✓	✠	✓	✓	✓
Iraq	2011	MICS	✓	✓	✓	✓	✓	✓
Jordan***	2012	DHS	✓	✓	✓	✓	✓	✓
Kenya	2008	DHS	✓	✓	✓	✓	✓	✓
Lao	2011	MICS	✓	✓	✓	✓	✓	✓
Lesotho	2009	DHS	✓	✓	✓	✓	✓	✓
Liberia	2013	DHS	✓	✓	✓	✓	✓	✓
Madagascar	2008	DHS	✓	✓	✓	✓	✓	✓
Malawi	2010	DHS	✓	✓	✓	✓	✓	✓
Maldives	2009	DHS	✓	✓	✓	✓	✓	✓
Mali	2012	DHS	✓	✓	✓	✓	✓	✓
Mauritania	2011	MICS	✓	✓	✓	✓	✓	✓
Mongolia***	2010	MICS	✓	✓	✓	✓	✓	✓
Morocco	2003	DHS	✓	✓	✓	✓	✓	✓
Mozambique	2011	DHS	✓	✓	✓	✓	✓	✓

Namibia	2013	DHS	✓	✓	✓	✓	✓	✓
Nepal	2011	DHS	✓	✓	✓	✓	✓	✓
Niger	2012	DHS	✓	✓	✓	✓	✓	✓
Nigeria	2013	DHS	✓	✓	✓	✓	✓	✓
Pakistan	2012	DHS	✓	✓	✓	✓	✓	✓
Philippines	2013	DHS	✓	✓	✓	✓	✓	✓
Rwanda	2010	DHS	✓	✓	✓	✓	✓	✓
S Tome & Principe	2008	DHS	✓	✓	✓	✓	✓	✓
Senegal	2014	DHS	✓	✓	✓	✓	✓	✓
Sierra Leone	2013	DHS	✓	✓	✓	✓	✓	✓
Somalia	2006	MICS	✓	✓	✓	✓	✓	✓
South Sudan	2010	MICS	✓	✓	✓	✓	✓	✓
State of Palestine***	2010	MICS	✓	✓	✓	✓	✓	✓
Sudan	2010	MICS	✓	✓	✓	✓	✓	✓
Swaziland	2010	MICS	✓	✓	✓	✓	✓	✓
Syria	2006	MICS	✓	✓	✓	✓	✓	✓
Tanzania	2010	DHS	✓	✓	✓	✓	✓	✓
Thailand***	2005	MICS	✓	✓	✓	✓	✓	✓
Timor-Leste	2009	DHS	✓	✓	✓	✓	✓	✓
Togo	2013	DHS	✓	✓	✓	✓	✓	✓
Tunisia***	2011	MICS	✓	✓	✓	✓	✓	✓
Uganda	2011	DHS	✓	✓	✓	✓	✓	✓
Vanuatu***	2007	MICS	✓	✓	✓	✓	✓	✓
Vietnam	2010	MICS	✓	✓	✓	✓	✓	✓
Yemen	2006	MICS	✓	✓	✓	✓	✓	✓
Zambia	2007	DHS	✓	✓	✓	✓	✓	✓
Zimbabwe	2014	MICS	✓	✓	✓	✓	✓	✓

✓	Data available
✠	Data available but it is not possible to recode original variable to follow standard definition for analyses
×	No data available
***	Countries excluded from the analyses as the number of women who delivered at home was less than 100
*year**	These surveys were replaced by previous surveys with information for all variables used in the analyses:
	Cambodia DHS 2010 was replaced by DHS 2000
	Indonesia DHS 2012 was replaced by DHS 2007

B)

World regions	Total number of countries by world region according to the UNICEF classification	Number of countries included in the analyses			
		National estimates	Stratified by wealth index	Stratified by maternal education	Stratified by area of residence
Eastern & Southern Africa	22	17	14	15	16
West & Central Africa	24	22	16	21	22
Middle East & North Africa	20	6	6	6	7
South Asia	8	7	6	6	6
East Asia & Pacific	27	6	5	6	5
Total	101	58	47	54	56
