# Supplementary material for: Who Delivers without Water? A Multi Country Analysis of Water and Sanitation in the Childbirth Environment
Source: PLoS One. 2016 Aug 17;11(8):e0160572. doi: 10.1371/journal.pone.0160572 (PMC4988668; doi:10.1371/journal.pone.0160572)
Supplement: S2 Table — (PDF) [file pone.0160572.s007.pdf]

Country	DHS, year	SPA, year
Kenya	2008-2009	2010
Rwanda	2010	2007
Uganda	2011	2007
Tanzania	2010	2006
