# Supplementary material for: Who Delivers without Water? A Multi Country Analysis of Water and Sanitation in the Childbirth Environment
Source: PLoS One. 2016 Aug 17;11(8):e0160572. doi: 10.1371/journal.pone.0160572 (PMC4988668; doi:10.1371/journal.pone.0160572)
Supplement: S3 Table — (PDF) [file pone.0160572.s008.pdf]

**Country**	**Hospital**	**Health centre**	**Dispensary**
Kenya	Hospitals	Maternity and health centres	Dispensaries and clinics
Rwanda	Hospitals	Health centres/polyclinic	Health post/dispensary/clinics
Uganda	Hospitals	HC-IV, HC-III	HC-II
Tanzania	Hospitals	Health Centre	Dispensary/Stand alone
