# Supplementary material for: Who Delivers without Water? A Multi Country Analysis of Water and Sanitation in the Childbirth Environment
Source: PLoS One. 2016 Aug 17;11(8):e0160572. doi: 10.1371/journal.pone.0160572 (PMC4988668; doi:10.1371/journal.pone.0160572)
Supplement: S4 Table — (PDF) [file pone.0160572.s009.pdf]

Type	Tanzania	Uganda	Rwanda	Kenya
**Respondent's own home**	Respondent's own home	Respondent's own home	Respondent's own home	Respondent's own home
**Other**	Other home, other	Other home, other, TBA, other public sector, other private sector	Other home, other, other public, other private	Other home, en route to provider, other public, other private medical, other
**Hospital**	Government (abbreviated to gov.) special or referral hospital, gov. regional hospital, gov. district hospital, specialised hospital (private), referral special hospital (religious), district hospital (religious)	Gov. hospital,	Gov. referral hospital, gov. district hospital	Gov. hospital, maternity
**Health centre**	Gov. health centre, health centre (private), health centre (religious)	Gov. health centre	Gov. health centre, private polyclinic	Gov. health centre
**Dispensary/ clinic**	Gov. dispensary or village health post, dispensary (private), dispensary (religious)	NA	Gov. health post, private clinic, private dispensary	Gov. dispensary
**Private**	NA	Private hospital or clinic	NA	NA
**Mission**	NA	NA	NA	Mission hospital or clinic/private hospital or clinic
