# Supplementary material for: Who Delivers without Water? A Multi Country Analysis of Water and Sanitation in the Childbirth Environment
Source: PLoS One. 2016 Aug 17;11(8):e0160572. doi: 10.1371/journal.pone.0160572 (PMC4988668; doi:10.1371/journal.pone.0160572)
Supplement: S5 Table — (PDF) [file pone.0160572.s010.pdf]

Tanzania

Regional classification in our dataset	SPA details	DHS details
Western	Tabora, Shinyanga, Kigoma	Tabora, Shinyanga, Kigoma
Northern	Kilimanjaro, Tanga, Arusha, Manyara	Kilimanjaro, Tanga, Arusha, Manyara
Central	Dodoma, Singida	Dodoma, Singida
Southern Highlands	Mbeya, Iringa, Rukwa	Mbeya, Iringa, Rukwa
Lake	Kagera, Mwanza, Mara	Kagera, Mwanza, Mara
Eastern	Dar es Salaam, Coast, Morogoro	Dar es Salaam, Pwani, Morogoro
Southern	Lindi, Mtwara, Ruvuma	Lindi, Mtwara, Ruvuma
Zanzibar	Unguja, Pemba	Unguja North, Unguja South, Town West, Pemba North, Pemba South

Uganda

Regional classification in our dataset	DHS	SPA
Kampala	Kampala	Kampala
Central	Central 1, Central 2	Central
East central	East central	East central
Eastern	Eastern	Eastern
North	North, Karamoja	North eastern (including most districts of Karamoja), North central
West Nile	West Nile	West Nile
Western	Western	Western
South West	South West	South West
