# Supplementary material for: Who Delivers without Water? A Multi Country Analysis of Water and Sanitation in the Childbirth Environment
Source: PLoS One. 2016 Aug 17;11(8):e0160572. doi: 10.1371/journal.pone.0160572 (PMC4988668; doi:10.1371/journal.pone.0160572)
Supplement: S6 Table — (PDF) [file pone.0160572.s011.pdf]

World region	Country	Unweighted N	Weighted N	Improved WATSAN		Improved water source		Improved sanitation facility	
				%	SE	%	SE	%	SE
Eastern & Southern Africa	Madagascar	6866	7125.0	0.1	0.0	24.2	1.6	0.3	0.1
	Tanzania	3455	3504.3	1.8	0.4	41.8	2.7	3.8	0.6
	Zambia	2843	2931.1	2.7	0.5	20.4	1.7	10.9	1.1
	Malawi	2588	2314.0	3.1	0.5	72.2	1.9	4.1	0.6
	Ethiopia	9293	9870.1	3.3	0.4	42.1	2.5	6.1	0.6
	Uganda	2558	2599.8	5.6	0.8	64.1	2.8	8.3	1.0
	Namibia	655	536.6	8.5	1.7	77.4	3.1	8.9	1.7
	South Sudan	3505	3480.7	8.6	1.0	68.6	1.8	11.1	1.1
	Kenya	3022	2883.0	9.3	1.5	49.4	3.4	13.4	1.7
	Somalia	1800	1777.3	11.2	1.5	19.7	2.3	28.6	2.4
	Lesotho	1387	1198.6	11.8	1.4	68.5	3.1	14.0	1.5
	Mozambique	288	288.0	14.9	2.8	51.9	5.0	20.7	3.3
	Comoros	674	665.4	18.4	2.5	75.3	4.2	22.3	3.1
	Burundi	2495	2762.6	24.1	1.8	72.1	2.3	32.4	2.2
	Zimbabwe	422	423.1	32.3	3.0	56.6	3.5	41.8	3.0
	Swaziland	141	145.8	34.0	4.5	52.6	4.3	58.1	5.2
	Rwanda	2434	2471.4	36.9	1.6	65.2	1.9	55.3	1.5
	**Mean**			13.3		54.2		20.0	
	**Median**			9.3		56.6		13.4	
West & Central Africa d	Benin	1635	1493.4	1.2	0.5	62.8	3.3	1.5	0.5
	Togo	2116	1746.7	1.3	0.3	40.0	3.1	1.6	0.4
	Congo Brazz	1141	545.3	1.4	0.7	27.1	3.8	2.0	0.9
	Congo DR	3890	2902.5	2.1	0.5	18.1	2.3	12.9	1.7
	Guinea Bissau	1307	1384.4	2.4	0.5	50.1	2.5	3.3	0.6
	Burkina Faso	4571	4965.3	3.3	0.6	61.9	2.2	3.9	0.6
	Niger	7692	8924.9	3.7	0.5	59.7	2.2	4.4	0.5
	Sierra Leone	3339	3531.6	3.9	0.6	43.3	3.0	6.3	0.8
	Cote d'Ivoire	3160	2822.5	5.8	0.8	64.9	3.4	6.7	0.9
	Liberia	2344	1685.3	6.0	1.0	58.1	3.5	6.9	1.1
	Gabon	934	339.3	6.4	1.5	70.8	3.3	6.5	1.5
	Mauritania	941	864.5	7.1	1.1	38.7	2.8	13.9	1.7
	Chad	4788	4968.4	7.2	0.9	46.0	2.6	8.6	1.0
	Mali	3876	4031.1	8.4	1.1	51.2	2.7	12.9	1.2
	Guinea	3598	3505.4	8.6	1.1	64.9	2.9	10.6	1.2

	CAR	1831	1796.2	10.6	1.4	50.5	2.9	15.7	1.5
	Senegal	1969	1313.7	15.4	2.2	60.4	4.7	19.2	2.4
	Cameroon	4044	4220.8	16.8	1.9	47.7	2.9	29.8	2.4
	S Tome & Principe	387	318.8	17.4	3.0	92.2	2.4	19.3	3.1
	Nigeria	17180	18043.8	20.7	1.5	47.5	2.1	33.6	1.7
	Ghana	1116	667.8	23.6	2.4	59.9	2.8	36.2	2.8
	Gambia	3303	2809.0	26.4	2.9	86.8	1.8	30.2	3.1
	**Mean**			9.1		54.7		13.0	
	**Median**			6.7		54.7		9.6	
Middle East & North Africa	Sudan	4508	4395.5	14.2	1.2	53.2	2.1	22.9	1.4
	Yemen	1140	1160.3	26.5	2.8	51.3	3.6	39.9	3.3
	Morocco	2508	2285.3	34.7	2.7	50.9	3.2	53.5	2.4
	Iraq	1539	976.1	70.5	2.2	72.9	2.1	93.4	1.0
	Syria	1049	1048.7	82.1	2.1	84.4	2.1	95.1	1.0
	Egypt	2958	2760.1	86.8	1.1	97.4	0.8	89.3	0.8
	**Mean**			52.5		68.4		65.7	
	**Median**			52.6		63.1		71.4	
South Asia	India	24640	28979.8	9.9	0.5	83.9	0.8	10.6	0.5
	Afghanistan	2633	2735.1	17.4	2.3	51.0	2.5	24.8	2.5
	Nepal	3253	3333.6	20.4	1.9	83.6	2.5	22.6	2.0
	Bangladesh	6341	6584.0	27.3	1.1	98.3	0.4	27.9	1.1
	Pakistan	5502	5671.3	28.4	2.4	52.8	3.5	43.8	2.8
	Bhutan	956	849.2	46.9	2.6	95.2	1.0	48.4	2.6
	Maldives	140	113.3	87.7	3.7	98.2	1.3	88.8	3.6
	**Mean**			34.0		80.4		38.1	
	**Median**			27.3		83.9		27.9	
East Asia & Pacific	Cambodia	7897	7162.6	2.4	0.3	26.7	1.9	6.8	0.7
	Vietnam	139	100.7	21.3	5.3	56.0	7.8	32.8	6.3
	Timor-Leste	7893	7623.6	24.5	1.3	59.0	1.7	34.2	1.4
	Lao	2705	2486.9	25.1	1.6	60.3	2.2	34.6	1.9
	Indonesia	10253	8228.0	25.2	1.2	51.3	1.6	40.4	1.4
	Philippines	2812	2489.6	45.8	1.7	88.5	1.3	49.1	1.7
	**Mean**			24.0		57.0		33.0	
	**Median**			24.8		57.5		34.4	
