# Supplementary material for: Who Delivers without Water? A Multi Country Analysis of Water and Sanitation in the Childbirth Environment
Source: PLoS One. 2016 Aug 17;11(8):e0160572. doi: 10.1371/journal.pone.0160572 (PMC4988668; doi:10.1371/journal.pone.0160572)
Supplement: S7 Table — (PDF) [file pone.0160572.s012.pdf]

	KENYA<br><br>%	TANZANIA<br><br>%	UGANDA<br><br>%	RWANDA<br><br>%
**NOT Piped**	56.5	63.5	79.7	37.4
**Source of water is NOT on site (within 500m)**	8.2	39.9	13.2	26.8
**Routine time of the year with a shortage of water**	46.1	52.0	49.5	60.2
**NOT improved in the delivery room**	16.1	52.3	29.2	39.8
